# Lead in School Children from Morelos, Mexico: Levels, Sources and Feasible Interventions

**DOI:** 10.3390/ijerph111212668

**Published:** 2014-12-08

**Authors:** Paulina Farías, Urinda Álamo-Hernández, Leonardo Mancilla-Sánchez, José Luis Texcalac-Sangrador, Leticia Carrizales-Yáñez, Horacio Riojas-Rodríguez

**Affiliations:** 1 Centro de Investigación en Salud Poblacional, Instituto Nacional de Salud Pública, Universidad No. 655 Colonia Santa María Ahuacatitlán, Cerrada Los Pinos y Caminera C.P., Cuernavaca, Morelos 62100, Mexico; E-Mails: paulina.farias@insp.mx (P.F.); leonardomancilla@yahoo.com.mx (L.M.-S.); jtexcalac@insp.mx (J.L.T.-S.); hriojas@insp.mx (H.R.-R.); 2 Departamento de Toxicología Ambiental, Facultad de Medicina, Universidad Autónoma de San Luis Potosí, San Luis Potosí 78000, Mexico; E-Mail: letcay@uaslp.mx

**Keywords:** Pb, lead sources, children, lead-glazed ceramics, Alpuyeca, Mexican candy

## Abstract

*Background*: Lead is a pervasive pollutant, associated at low levels to many adverse health effects. *Objective*: To investigate lead levels, exposure pathways and intervention possibilities in school children from Alpuyeca, in Morelos, Mexico. *Methods*: Blood lead concentrations (BPb) were measured in 226 children in 2011. Exposure pathways were assessed through a questionnaire, lead measurements in different environmental matrices and spatial aggregation analysis of lead concentrations. *Results*: BPb ranged from 1.5 to 36.5 µg/dL, with a mean (SD) of 7.23 (4.9) µg/dL. Sixty-four and 18% of the children had BPb > 5 µg/dL and > 10 µg/dL, respectively. The use of lead glazed ceramics was reported in almost half of the households; it was the main BPb determinant and it was associated with an increased risk of having BPb > 5 g/dL by 2.7 times (*p* = 0.001). Environmental samples were within US EPA’s lead recommended limits, and blood lead levels were randomly distributed in the community. *Conclusions*: Lead remains a public health problem in Alpuyeca, Mexico. Unlike other local pollutants, lead exposure prevention can be achieved inexpensively and in a short term. Interventions should make mothers aware of lead’s health effects and empower them to safeguard their children’s health by avoiding the culturally ingrained use of lead glazed pottery.

## 1. Introduction

Even though lead has been widely studied, findings associating low concentrations of this pollutant to health effects (especially in children’s nervous systems) [1,2] justify monitoring its levels and preventing its exposure as much as possible. Sources of lead exposure and blood lead (BPb) levels have been diminishing in Mexico and the world over the years [3,4], but so have the blood lead levels considered acceptable [5]. Although lead is present worldwide, its sources may vary in different places. Lead pollution can come from former industrial sites or active industry, such as mining, battery recycling plants and smelters [6,7]. Other sources in developed countries are mainly old lead-based peeled or chipped paint, especially present during renovations of old houses [8,9] and contact with contaminated dust or soil [10]. Some developing countries also experience exposure through the use of leaded gasoline [11], toys and products for babies or children containing paint with lead, traditional medicines or remedies, cosmetics and the consumption of local candies containing lead [12,13]. In Mexico and other countries, the use of lead glazed ceramics is culturally ingrained and is currently the main exposure source to lead [14,15,16].

Unlike other metals, lead is purely toxic. It has neither a known function in the human body nor a recognizable threshold [17,18,19]. However, it does represent about 0.6% of the global burden of disease [20]. Toxic effects of lead in children can go from subclinical, but important ones [21], at doses lower than 5 µg/dL of blood lead (CDC reference level to initiate public health actions) to deadly toxic effects at doses around 150 µg/dL of BPb [19]. Every organ system in the body can potentially be affected by lead, the main ones being the nervous [22] cardiovascular [21], renal [23], endocrine [24], immune [25] and hematological [26] systems.

Children are especially vulnerable regarding lead. On the one hand, children are more likely to be exposed. Their behavior may increase lead exposure by hand to mouth ingestion of lead in soil and dust or by pica (eating non-food materials that can be contaminated) [27]. They are also prone to having a greater intake and burden than adults due to: a higher inhalation rate relative to body mass; more intestinal absorption (50% in children *vs.* 10% in adults), and six times more retention of absorbed lead. On the other hand, lead can produce more severe and permanent effects in children because of its interference with developmental events [20,28].

Furthermore, dietary deficiencies faced by children living in poverty, enhance lead’s absorption and/or effects. Low calcium, iron, zinc and vitamin C intake are examples of nutrients that influence lead’s availability and toxicity [29,30,31].

The children in this study lived in Alpuyeca, a small town in Morelos, Mexico, facing complex environmental pollution problems. There is an open-air sanitary landfill 6 km away, an industrial landfill containing polychlorinated biphenyls (PCBs) near the downtown elementary school, and exposed animal fecal matter all over. According to the 2010 Mexican national census, Alpuyeca was classified as having a high degree on marginalization [32]. This study investigated blood lead levels and exposure routes, along with possible interventions to diminish the former and prevent the latter in school children from Alpuyeca.

## 2. Methods

### 2.1. Study Design

This is a cross sectional study which is part of a larger one aimed at studying a complex environmental situation in which several pollutants and poor sanitation interact in Alpuyeca, Morelos [33].

### 2.2. Recruitment of Participants

In 2011, parents and children from two public schools, including both the morning and the afternoon shifts, were briefed about the study. Those who agreed to participate signed an informed consent form, and thus, a sample by convenience of 226 children was obtained.

### 2.3. Questionnaire

A questionnaire on lead exposure sources, socio demographic characteristics and dietary habits was applied to all participants’ mothers. This questionnaire was especially designed for Alpuyeca residents. It was based on a questionnaire previously developed and applied in a similar study in the town of Tlamacazapa, Morelos [34]. On February 2011, the questionnaire was tested in ten mothers from Atlacholoaya, a community near and very similar to Alpuyeca. Necessary modifications to the questionnaire were identified: specifying not only if tap water was available, but whether the faucet was inside or outside the house and adding the response option “doesn’t know” for every question. The modified questionnaire was evaluated again in six more mothers and we confirmed it was clear and complete.

### 2.4. Laboratory Analysis

Blood samples of approximately 10 mL were drawn from each and every participating child to measure blood lead and hemoglobin levels. Three laboratory technicians drew the blood samples through venipuncture at the schools. All materials used were new and disposable. The blood sample was divided into two separate tubes for each participant, lead-free and containing ethylenediaminetetraacetic acid (EDTA) as anticoagulant. During the sampling, all samples were kept in coolers with ice packs. One of the tubes of each participant was immediately transported to a local laboratory for hemoglobin analysis. The other tube of each participant was transported to the Center for the Research of Infectious Diseases at the National Institute of Public Health, Mexico, were it was stored in freezers at 4 °C. The latter tubes were then shipped to the Environmental Health Laboratory of the Environmental Toxicology Laboratory at the Autonomous University of San Luis Potosí, Mexico to determine lead levels. Concentrations of blood lead were determined through atomic absorption spectroscopy using the method described by Subramanian [35] with matrix modifiers (DMH-Triton X-100 in nitric acid at 0.2%). The equipment used was a 3110 Perkin Elmer spectrometer with a graphite furnace.

### 2.5. Determination of Lead in the Environment

New pieces of both non-glazed and glazed ceramics were purchased at local markets to measure their lead levels by analyzing them through two methods. Three pieces of glazed ceramics and three pieces of non-glazed ceramics were analyzed by each method. The first method, migration test, consisted of exposing the inner surface of the ceramic vessels to 4% acetic acid at room temperature for a period of 24 h. Vessels were filled with the solution, kept away from the light, and covered with a glass lid to avoid contamination and evaporation. After the 24 h period, the solution was stirred with a glass rod and lead levels in it were determined through atomic absorption spectrophotometry.

Lead was also measured in ceramic cookware through graphite furnace atomic absorption spectroscopy by completely grinding ceramics. Three samples were taken of each ground piece of cookware. The dust of the samples was digested by the microwave extraction technique.

We also studied six kinds of candy, representing those most frequently consumed by children in Alpuyeca. Ten pieces of each kind of candy were collected and three samples of each piece were analyzed. The candy samples were digested by the microwave extraction technique and their lead content was determined through graphite furnace atomic absorption spectroscopy.

Both ceramics and candy were analyzed by the Laboratory of the Department of Ecology and Natural Resources of the Biology School of the National Autonomous University of Mexico.

Three different water sources were tested: from the local river at a recreational spot (three samples), from participants’ drinking water source (bottled water in all cases) and from stored tap water that is pumped from the local well (Alpuyeca inhabitants receive running water for limited periods of time each week and store it in basins). Water was sampled in 17 homes, corresponding to those children with the highest BPb levels. One liter was collected from each source. All water samples were analyzed by the Laboratory of Research and Development for Water Quality Studies (IDECA) in Mexico for lead measurements. This laboratory specializes in environmental monitoring and is accredited by the Mexican Entity of Accreditation, Civil Association. Lead was measured by atomic absorption spectroscopy according to the Mexican Norm [36].

Sediment, soil, and dust lead levels were determined by the Mexican Institute of Water Technology (IMTA) through atomic absorption spectroscopy [37].

Dust samples were obtained from the interior windowsills in the child’s bedroom and from the top of their wardrobe using dust wipes.

Superficial soil was sampled from: the playgrounds in both elementary schools (three sampling points where children play the most in each one), 13 homes of children with the highest BPb levels (one sample of the front or back yard, depending on what they had or where the child usually played), and in a recreational area next to the river (three samples taken). Soil samples were collected by first removing surface debris, such as leaves, sticks, stones, *etc.* at the sampling site. Then, the soil was swept to form a small mound. Using an aluminum spoon, the soil from the mound was mixed by scooping from the bottom layers to the top and vice versa. The mixed soil was then scooped into a one quart, resealable plastic bag that had been previously labelled with its corresponding code, site, date and time.

Sediment samples were obtained from the river and from the dam, three samples in each site. The technique consisted of digging a 30 cm. deep hole with a metal spade. The sediment was then scooped with an aluminum spoon into plastic bags as previously described for soil.

### 2.6. Statistical Analysis

Stata 11 software [38] was used to perform the statistical analysis of the data. All the information from the questionnaire and laboratory results was captured electronically. Blood lead levels, the dependent variable, was originally continuous, but it was also dichotomized at two cut-off points of public health relevance: >5 and >10 µg/dL. A dichotomous variable to explore potential para-occupational exposure was developed. If either one or both parents worked in the following occupations or activities: mechanic, construction worker, painter, pesticide applier, scavenger, driver, or traffic police officer, potential para-occupational exposure to lead was considered present.

A descriptive analysis, both numerical and graphical, was conducted on all variables to determine their shape and distribution. The relationship between independent variables with each type of blood lead variable was then tested. *T*-tests were carried out to compare mean blood lead levels of each category in dichotomous independent variables. Chi-squared tests were used for categorical independent variables and correlations and linear regressions were used for continuous independent variables. Since the BPb levels variable had a skewed to the right distribution, it was log-transformed whenever it was analyzed in linear regressions to comply with the normal distribution assumption of the dependent variable in this analysis. Multivariate analyses were performed using linear regression for lead as a continuous variable and logistic regression for lead as a dichotomous variable. Initial regression models in each case were created introducing all independent variables that had shown bivariate associations to lead (*p* ≤ 0.2) or had biologically plausible relations. These models were adjusted by age, sex and body mass index (BMI). Independent variables were then excluded one at a time from the models if they did not reach a statistical significance of *p* ≤ 0.05, unless they influenced the rest of the coefficients by more than 10% their value.

The population sample originally recruited for this study included eight pairs of siblings and two trios of siblings. Since some statistical tests applied, such as linear regressions, assume independence among observations, only the youngest sibling in each family was kept in the sample and the rest were discarded. In other words, the eight older siblings from the eight pairs were eliminated and the two older sibling of each of the two trios were eliminated for a total of 12 children eliminated from analysis.

### 2.7. Geo Spatial Analysis

Each participant’s home was geographically referenced using a global positioning system (GPS) eTrex^®^ Legend (Garmin Ltd., Olathe, KS, USA), which had an accuracy of ±3 m. An exploratory analysis was conducted on spatial data in order to identify possible aggregations of subjects with higher blood lead levels in certain parts of the study area. The statistics used to evaluate spatial correlations were: Moran I, as a global method, and the Local Indicator of Spatial Association (LISA), as a local method [39,40].

### 2.8. Study Approval

All committees of Ethics, Research and, Biosecurity of the National Institute of Public Health approved this project.

## 3. Results

The final population sample consisted of 226 children, of whom 119 were girls and 107 were boys. The children’s age ranged from six to 13 years, with a median of ten. These children were almost evenly distributed in school grades first to sixth.

Participants came from different neighborhoods. Two of these neighborhoods are considered less marginalized than the rest, and almost half of the children lived there.

Only 5% of children had hemoglobin levels ≤11 g/dL, the lower normal limit for this age group, and the lowest hemoglobin value was 10 g/dL. Blood lead levels went from 1.5 to 36.5 µg/dL, with a mean (SD) of 7.23 (4.9) µg/dL. When we did take into account the twelve siblings that were eliminated from the analyses to comply with the assumption of independence of values, the range of blood lead levels did not change, and the mean(SD) was not significantly different: 7.16 (4.8) µg/dL. Approximately 64 and 18% of the children had blood lead levels above five and 10 µg/dL, respectively.

Median and mean differences in BPb levels by dichotomous characteristics of participants or potential lead sources were explored. Using the Wilcoxon Mann-Whitney test, the only significant difference found was between median BPb levels of those who used lead glazed ceramics and those who did not (P(z) = 0.003). Mean BPb differences between presenting and not presenting a potential source or risk factor are described in Table 1.

**Table 1:** Comparison of mean blood lead levels by presence *versus* absence of potential sources and risk factors in school children from Alpuyeca, Mexico using *T*-test.

Potential Sources or Risk Factors for Higher Blood Lead Levels (%)	Mean Blood Lead Level (µg/dL)	Mean Blood Lead Difference Comparing to Reference Group (µg/dL)	*T*-Test P(t)
Male sex (47)	7.57	0.69	0.29
Exposed to second-hand smoke at home (37)	6.82	−0.69	0.31
Food is cooked in lead glazed ceramics at home (48)	7.98	1.4	0.03
Food is stored in lead glazed ceramics at home (12)	9.29	2.39	0.02
Eats potentially lead-contaminated candies (84)	7.29	0.38	0.66
Pica (soil, paint, pencils or chalk) (55)	7.23	0.01	0.99
No flooring within the house (bare soil) (26)	8.00	1.04	0.16
Painted walls in the house (47)	6.55	−1.34	0.05
No tap water available in the house (53)	7.06	−0.4	0.55
Home located in poorest neighborhoods (53)	7.28	0.11	0.87
Attends afternoon school shift (56)	7.65	1.55	0.28
Possible para-occupational exposure to lead (18)	7.02	−0.26	0.76

Both age and school grade were negatively correlated to BPb, however these correlations were not significant: r = −0.044 (*p* = 0.52) and r = −0.55 (*p* = 0.42), respectively. Neither was a significant association found with BPb when age and school grade were categorized.

After creating a multivariate model for the log-transformed BPb levels as a dependent variable and eliminating step by step independent variables that were not significant, only the use of lead glazed ceramics (yes *vs.* no) remained in the model in the end (coefficient = 0.24 and 95% CI = 0.08, 0.39).

Blood lead levels ≥5 µg/dL, were also significantly associated to the use of lead glazed ceramics, with an odds ratio of 2.68 and a 95% CI = 1.52, 4.72. This was the only variable significantly associated to having BPb levels ≥5 µg/dL. The analysis of associations between lead levels dichotomized at 10 µg/dL yielded no significant results, not even with the use of lead glazed ceramics.

Results of lead measured in potential local sources, such as ceramics, candies, and relevant media: soil, sediment and water, are shown in Table 2. Only one type of candy, “Huevines” (candy coated chocolate eggs), had lead levels almost twice above the recommended FDA limit.

**Table 2:** Range and mean (SD) of lead levels measured in different media in Alpuyeca, Morelos and the corresponding local and/or international lead reference values for each medium.

Sampled Medium	Location and Number of Samples	Sampling Date	Lead Concentrations in Medium		Reference Values of Medium
			Range	Mean (SD)	
**Soil**	Soccer field in downtown elementary school (N = 3)	April 2011	5.73, 54.43 mg/kg	28.80 (15.3) mg/kg	Mexican Official Norm for agricultural, commercial and residential use = 400 mg/kg [41]
	Playground in both elementary schools (N = 3)	April 2012	10.95, 46.62 mg/kg	24.69 (13.7) mg/kg	EPA for play areas= 400 mg/kg (US EPA, 2001) [42]
	Front or back yard of homes corresponding to children with the highest BPb levels (N = 13)	April 2012	8.22, 37.47 mg/kg	18.05 (8.1) mg/kg	ATSDR defines non-polluted soil <50 ppm [43]
**Dust**	Windowsills and furniture of homes corresponding to children with the highest BPb levels (N = 13)	April 2012	10.85, 99.69 mg/kg	43.16 (31.9)	Not available
**Sediment**	Alpuyeca’s dam (N = 3)	April 2012	1.97–17.24 mg/kg	12.32 (4.7) mg/kg	Not available
	Alpuyeca’s river (N = 3)	April 2012	6.09–7.32 mg/kg	6.77 (0.6) mg/kg	
**Water**	Stored tap water from study homes (N = 17), bottled water (N = 17) and superficial water in a recreational area of the river (N = 3)	April 2011	All below detection limit = 0.005 mg/L	below detection limit	EPA’s action level = 0.015 mg/L [44]
					Mexican Official Norm for water use and consumption (NOM-127-SSA1-1994) = 0.01 mg/L [45]
**Non-glazed ceramics**	Cuentepec street market (N = 3)	October 2012	Detection limit: 0.04 mg/Kg	8.75 (0.04) mg/Kg of total lead	Not available
	Cuentepec street market (N = 3)	December 2012	All below detection limit: <0.010 mg Pb/L acetic acid solution	Below detection limit: <0.010 mg Pb/L acetic acid solution	Mexican Official Norm for glazed ceramics= 0.5 Pb to 2 mg/L acetic acid solution [46]
**Glazed ceramics**	Alpuyeca street market (N = 3)	October 2012	Detection limit: 0.04 mg/Kg	27.50 (0.06) mg/Kg of total lead	Not available
	Alpuyeca street market (N = 3)	December 2012	38.23, 278.72 mgPb/L acetic acid solution	198.19 mg/L	Mexican Official Norm for glazed ceramics= 0.5 to 2 mg Pb/L acetic acid solution [46]
**Local candy**	Six kinds of candy, ten pieces of each (N = 60)	October 2012	<0.004 (detection limit), 0.176 ppm	0.034 (0.01) ppm	FDA recommended maximum lead level of 0.1 ppm in candy likely to be consumed frequently by small children [47]

**Figure 1:**
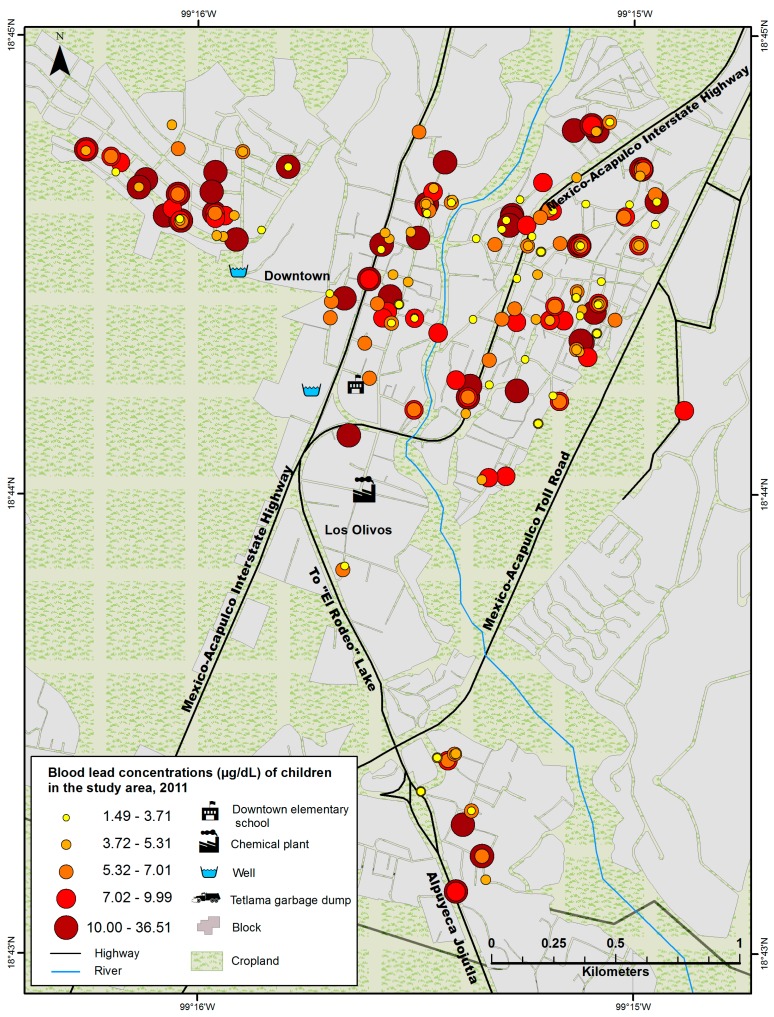
Geographic distribution of blood lead concentrations in school children from Alpuyeca, Morelos according to home location.

No association was found between BPb concentrations and residence location of study subjects. Neither the global nor the local analysis of Moran’s I coefficients showed a significant association between specific locations in the study area and BPb levels. Further exploration of spatial aggregation analysis to detect a specific environmental source of lead was not possible since BPb concentrations and residence showed no aggregation or disaggregation pattern. This means BPb levels in the study area were randomly distributed (Figure 1).

## 4. Discussion

In this study, lead levels in glazed ceramics were on average 100 times higher than what is accepted by the Mexican Official Norm, emphasizing the importance of this source of exposure. Even though ceramics samples, glazed and non-glazed, were obtained from local markets and not from participants’ homes, it is probably safe to assume that participants get their ceramics from those markets. Few ceramics samples were analyzed and they might not have been representative of what all participants use. However, the association between the use of lead glazed ceramics, as referred by mothers of participants, and BPb holds valid.

Dichotomous factors unlikely to be lead sources, because they were not associated to lead levels of children in this study, were: potential para-occupational exposure (coefficient = −0.099, CI95% = −0.30, 0.10), second-hand smoke (coefficient = −0.099, CI95% = −0.26, 0.06), pica (coefficient = 0.02, CI95% = −0.14, 0.17), or eating local candies (coefficient = 0.02, CI95% = −0.15, 0.19). Measurements of lead levels in the most frequently consumed candies in Alpuyeca suggest this is not generally a source of lead exposure. However, candy wrappers were not tested for lead levels and even if the candy itself did not contain lead, there remains a possibility that wrappers were printed with lead-based ink and contributed in some degree to BPb levels. We do not know the amount or frequency of consumption of the one specific type of candy that showed higher than FDA accepted levels, but the statistical analysis suggests it is not affecting these children’s BPb levels. Nonetheless, it must be made known that “Huevines” should be avoided by children everywhere. In fact, the California Department of Public Health had already detected high lead levels in these candy coated chocolate eggs and issued a warning to avoid them since 2008 [48].

The random distribution of blood lead levels in the study area, as seen in the geospatial analysis, further corroborates there is no environmental source that significantly contributes to the children’s exposure to lead. Supporting these findings, measured lead levels were not above values considered acceptable by Mexico and the EPA in the following places: where participants live; where they play at school or at home; in their tap water; in the river where they swim and in the local dam.

Hemoglobin levels were mostly in a normal range for the children’s age, indicating there is no important iron deficiency in these children’s diet in general. Therefore, we see no need to recommend iron supplementation. However, recommendations for a well-balanced diet (including enough calcium, zinc, vitamin C, and iron) could help.

This study has the advantage of supporting the findings regarding sources of lead (whether present or absent), detected through BPb levels’ statistical associations to determinants explored through a questionnaire, with direct measurements of lead in several matrices and with geospatial analysis.

Since the use of lead glazed ceramics has been well documented as the main source of lead exposure in Mexico after lead was definitely removed from gasoline since 1996 [15,49], alternatives to abate this source have been tried. Some of these alternatives have included the exchange of traditional kilns with high- temperature kilns (1200–1800 °C), gas-fired kilns and the use of lead-free glaze. The United States Agency for International Development (USAID), the World Bank and the Mexican government agency, Fondo Nacional para el Fomento de las Artesanías (FONART), have subsidized gas kilns. Even though this is a laudable measure, it is nowhere near to solving the problem. Gas-fired kiln manufacture is expensive, the costs per kiln is around US $20,000. Only some 48 kilns have been constructed by FONART through subsidies, and there are approximately 50,000 ceramic producers [50]. The use of lead-free glazes has not been largely adopted either, only around 3% of the pottery produced in Mexico is lead-free. Lead-free glazes produced with oxides of: zinc, lithium, sodium, potassium, barium, calcium, strontium, magnesium, beryllium and boron, have characteristics that are not always accepted by producers and/or consumers. For instance, they are not as shiny or have a yellowish tone. Artisans have reported losses of up to 40% of their sales when using lead-free glazes [51,52].

Lead damages health and thus, places an economic burden on families and society. A cost-benefit analysis found that for every dollar spent to reduce lead hazards in the United States, there is a benefit of 17 to 220 dollars. This cost–benefit ratio is even better than that for vaccines, which have been considered the single most cost-beneficial medical or public health intervention [53]. Another study in France concludes that lowering BPb levels below 1.5 μg/dL or 2.4 μg/dL would have overall annual benefits in 2008 of €22.72 billion and €10.72 billion, respectively [54]. Preventing lead exposure in Alpuyeca and in communities in a similar situation might have a greater cost-benefit ratio than in other more developed places, since BPb levels are higher and the single main source (use of lead glazed ceramics) could be targeted more easily and eliminated more quickly than other sources (removing leaded paint and old water pipes) and at a much lesser cost.

Achieving a decrease of BPb levels by eliminating the use of lead glazed ceramics would perhaps not only avoid health effects directly associated to lead, but also possible effects resulting from the interaction between lead and other pollutants, such as PCBs present in the area and neurodevelopment alterations. A plausible intervention that can make a difference is a well planned and carried out risk communication involving local mothers. If mothers are made aware of lead contents in lead glazed ceramics and lead exposure and effects in children, they can be empowered to find alternatives and protect their children’s health.

## 5. Conclusions

The fact that most children studied have BPb levels above what has been associated to health effects and above what the CDC considers necessary to start public health actions, indicates that lead is still a problem in Alpuyeca, Morelos in Mexico. Not only were 145 children above CDC’s 5 µg/dL level of action, 42 children are above Mexico’s acceptable level of 10 µg/dL.

Results in this study consistently point to the same principal source of lead exposure in Alpuyeca: the use of lead glazed ceramics. In the end, the only significant association between lead levels, continuous or dichotomized at 5 µg/dL, was the use of lead glazed ceramics. The magnitude of the association was important, considering that the use of lead glazed ceramics increased 2.7 times the probability of a child having BPb levels above 5 µg/dL.

As with any other health problem, prevention is the best way to deal with lead exposure and its effects. The challenge in this case arises mostly from changing a culturally ingrained habit: the use of lead glazed ceramics for cooking, serving and storing food. Lead glazed ceramics is an affordable cookware, people are used to it, they see it as part of the Mexican culture and they prefer the taste of food cooked in these utensils. People also argue they have been using lead glazed ceramics for generations and nothing has ever happened to them. Nonetheless, unlike other pollution problems in Alpuyeca, diminishing lead exposure is feasible and can be achieved in a short term. Interventions with ample community participation are already being implemented in Alpuyeca. These interventions include risk communication and the promotion of non-glazed local ceramics use. Results will be published in the following months.

Findings of this study must be spread to the scientific community and to other communities (such as artisans) in Mexico and in the world where lead glazed ceramics is used in order to help solve a public health problem that is not new, but that is still prevalent and preventable.
